# Wistar rats immature testicular tissue vitrification and heterotopic grafting

**DOI:** 10.5935/1518-0557.20180023

**Published:** 2018

**Authors:** Larissa Benvenutti, Rafael Alonso Salvador, David Til, Alfred Paul Senn, David Rivero Tames, Nicole Louise Lângaro Amaral, Vera Lúcia Lângaro Amaral

**Affiliations:** 1 Laboratório de Biotecnologia da Reprodução (LBR), Universidade do Vale do Itajaí (UNIVALI), Itajaí, Santa Catarina, Brazil; 2 Centro Clínico Veterinário (CCV), Itajaí, Santa Catarina, Brazil

**Keywords:** cryopreservation, prepubertal, fertility preservation, transplantation

## Abstract

**Objective:**

To evaluate the efficiency of two vitrification protocols for rat immature testicular tissue and heterotopic transplantation.

**Methods:**

Twenty-four pre-pubertal Wistar rats were divided into three groups (n=8). After orchiectomy, testicular fragments (3mm) from Groups 1 and 2 were vitrified with different cryoprotectant concentration solutions, using sterile inoculation loops as support. After warming up, the fragments were submitted to cell viability assessment by Trypan blue and histological evaluation. Vitrified (Groups 1 and 2) and fresh (Group 3) fragments were grafted to the animals periauricular region. After 8 weeks of grafting, the implant site was histologically analyzed.

**Results:**

The viability recovery rate from Group 1 (72.09%) was higher (*p*=0.02) than that from Group 2 (59.19%). Histological analysis showed similar tubular integrity between fresh fragments from Groups 1 and 3. Group 2 samples presented lower tubular integrity. We ran histological analyses in the grafts from the Groups. In all groups, it was possible to see the implant site, however, no fragment of testicular tissue or signs of inflammation were histologically found in most samples from Groups 1 and 3. In one sample from Group 2, we found degenerated seminiferous tubules with necrosis and signs of an inflammatory process. In another sample from Group 2, we found seminiferous tubules in the implant site.

**Conclusion:**

The vitrification of pre-pubertal testicular tissue of rats showed little damage to cell viability through histological analysis when we used cryoprotectants in a lower concentration. Heterotopic transplantation could not preserve the structural organization of the testicular tissue.

## INTRODUCTION

Infertility is considered a reproductive system disease that consists of an absence of clinical pregnancy after 12 months of unprotected intercourse (Jose-Miller *et al*., 2007). There are several causes for male infertility, which include gonadotoxic cancer treatments, such as chemotherapy and radiotherapy. Such treatments may damage somatic cells, such as Sertoli cells and germ cells, which can result in temporary or permanent infertility (Wallace *et al*., 2011). The fact that usual treatments for cancer lead to male infertility makes strategies such as semen cryopreservation in adult men an approach to preserve these patient's fertility. Prepubertal boys, however, do not benefit from sperm banking, and they are subjected to the same deleterious effects of gonadotoxic treatments, making their fertility preservation a challenge (Brinster, 2007).

One potential alternative for fertility preservation of these boys is testicular tissue cryopreservation, as fragments (Brinster, 2007) or cell suspensions (Yango *et al.*, 2014). It has been suggested the grafting of cryopreserved testicular fragments, where the germ cells could differentiate and eventually produce spermatozoa (Brinster, 2007). However, this technique is still considered experimental and with varied results in testicular tissue samples. Orthotopic and heterotopic transplantations were successfully performed on ovarian fragments, with reports of baby births after tissue cryopreservation and transplantation (Sánchez-Serrano *et al*., 2010; Silber, 2012).

Studies in mice (Wu *et al*., 2012), pigs (Kaneko *et al*., 2014) and even humans (Unni *et al*., 2012; Yango *et al*., 2014), demonstrate the efficiency of slow testicular tissue cryopreservation in preserving cell viability. Vitrification is a cryopreservation strategy that differs from slow cryopreservation due a vitreous state formation that provides a sufficient high cooling to the cells forming only extracellular ice (Pegg, 2005), which would theoretically prevent ice crystal damage, which happens in slow cryopreservation. Vitrification has emerged as a promising method to cryopreserve ovarian tissue (Curaba *et al*., 2011; Herraiz *et al*., 2014) and it is poorly reported in the literature as immature testicular tissue cryopreservation (Curaba *et al*., 2011; Poels *et al*., 2012).

In order to preserve cell survival after storage at low and stable temperatures, cryoprotectants are used to reduce the chemical damage caused by freezing in slow cryopreservation and vitrification (Woods *et al*., 2004; Rubinsky *et al*., 2005). Cryoprotectants used in cryopreservation methods, however, can be potentially toxic to the cells (Woods *et al*., 2004). Dimethyl Sulfoxide (DMSO) and Ethylene Glycol (EG) cryoprotectants appear to be less toxic in slow cryopreservation of prepubertal testicular tissue (Unni *et al*., 2012). The aim of this study was to evaluate the efficiency of two distinct vitrification solutions with different cryoprotectant concentrations in preserving cell viability and assessing heterotopic grafting results of vitrified testicular fragments.

## MATERIALS AND METHODS

After obtaining the approval from the Ethics Committee in Animal Use (ECAU) of the Vale do Itajaí University through the 001/16 protocol, 24 male Wistar rats in prepubertal age of approximately 25 days old, were randomly divided in three groups: Group 1 (Vitrification protocol 1), Group 2 (Vitrification protocol 2) and Group 3 (Control group).

### Orchiectomy

For the orchiectomy, the animals were anesthetized with acepromazine 1% (2/kg), ketamine chloride (35/kg) and xylazine chloride 2% (5/kg) association diluted in injection water (Schnaider *et al*., 2002). The testes were removed by a scrotal incision; and after the surgical procedure the testes were fragmented in pieces of approximately 3mm and used as autologous graft into the periauricular region, immediately after the orchiectomy (Group 3) or were subjected to vitrification protocols (Groups 1 and 2). After the surgical procedure, two doses of subcutaneous analgesic 1% ketoprofen (5mg/kg) within a 24h interval (Lu *et al*., 2004) was administrated to each animal.

### Cell Viability Assessment

Cell viability of fresh (Groups 1, 2 and 3) and post-vitrification (Groups 1 and 2) fragments was assessed by Trypan Blue Exclusion Assay, using 10 µL of Trypan Blue vital staining (Sigma-Aldrich, Saint Louis, MO, USA) for each 10µL of digested fragment, enzymatically digested by Trypsin (0.05%) and Hyaluronidase (1:1) for 5 minutes. The cells were categorized as viable (not stained) and non-viable (stained) (Yango *et al*., 2014).

### Testicular Tissue Vitrification

The Group 1 testicular fragments were vitrified with Ingámed^Ⓡ^ vitrification commercial kit. The tissue was initially treated with an equilibrium solution (VS1) made up of 7.5% DMSO and 7.5% EG for 10 minutes at 4ºC. The fragments were then transferred to a vitrification solution constituted of 15% EG, 15% DMSO and 0.5 M sucrose, remaining there for 5 minutes at 4ºC (Borges Júnior *et al*., 2014).

The Group 2 testicular samples were vitrified with the Santos *et al*. (2007) vitrification solution and the fragments were first exposed to an equilibrium solution constituted of 10% of DMSO, 10% of EG, Sucrose 0,25M e HTF (Human Tube Fluid)-Modified (Irvine^Ⓡ^) + 10% SFB for 7 minutes at 4ºC. Later, the fragments were transferred to a vitrification solution composed of 20% DMSO, 20% EG, 0,5M Sucrose e HTF-Modified + 10% SFB for 3 minutes at 4ºC.

Fragments from both groups were then transferred to a sterile inoculation loop of 1µL (Olen^Ⓡ^) that served as support, and were immersed into precooled cryovials (KASVI^Ⓡ^) with liquid nitrogen (LN2). Cryovials were closed and stored in LN2 for 60 days.

### Warming

Group 1 testicular fragments were retrieved from the cryovials and immersed in Ingámed^Ⓡ^ warming solution (DV1), exposed for 1 minute at 37ºC. The fragments were then transferred to a warming solution (DV2) for 5 minutes, proceeded by a warming solution (DV3) for another 5 minutes (Borges Júnior *et al*., 2014). The samples were then subjected to cell viability assessment or grafted into the periauricular region.

The Group 2 testicular fragments were immersed in HTF-modified (Irvine Scientific) + SFB + 1M sucrose for 1 minute at 37ºC, and were exposed to solutions with decreasing sucrose concentration right after (0.5 M, 0.3 M) for 5 minutes each. The fragments were then transferred to an HTF-modified (Irvine Scientific) + SFB for 5 minutes (Poels *et al*., 2012). The fragments were subjected to cell viability assessment, histological analysis or grafted into the periauricular region.

### Histological analysis of the testicular fragments

The fragments were fixed directly in 4% formaldehyde, embedded in paraffin, and then cut into serial sections of 5µm thickness for histological evaluation. The sections were fixed in hematoxylin-eosin for optical microscope analysis (Poels *et al*., 2012).

Ten fragments from seminiferous tubules from each sample from the three groups were analyzed at 1000x magnification in cross sections. For each seminiferous tubule, tubular integrity was measured using a score of 0-4.

A point was given for each parameter: no basal membrane detachment, visualization of spermatogonial nuclei, identification of germ cells and Sertoli cells, and absence of hyalinization (Curaba *et al*., 2011; Lima *et al*., 2017).

### Testicular Grafting

For testicular tissue grafting, the animals were anesthetized and the fragments were grafted through an autologous transplant in the periauricular region of the animals. An incision in the periauricular region was made with a bistoury and the region was divulsed for the introduction of the fragment, with post cauterization of the incision. Group 3 received the fragments right after the orchiectomy and Groups 1 and 2 received the grafts post-vitrification and warming, two weeks after the orchiectomy.

The animals were euthanized in a CO_2_/O_2_ chamber, 60 days after the transplant, and grafting site histology was performed.

### Statistical Analysis

For viability purposes, we ran a statistical analysis of variance using ANOVA with Tukey's comparison post-test. A *p*<0.05 was considered statistically significant.

## RESULTS

### Cell viability after tissue cryopreservation

**Figure 1:**
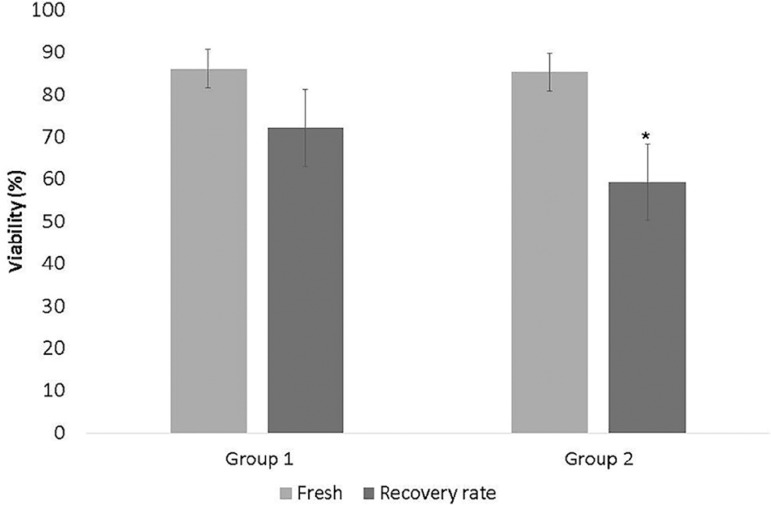
Cell viability recovery rate after vitrification. * Significantly different from group 1 recovery rate (*p*<0.05)

Figure 1 depicts cell viability of fresh fragments and after vitrification and warming. Vitrified samples from Group 1 presented a higher recovery rate of 72.09±9.13 (*p*=0.02) than those from Group 2 (59.19±10.58).

### Testicular fragments histological analysis

**Figure 2:**
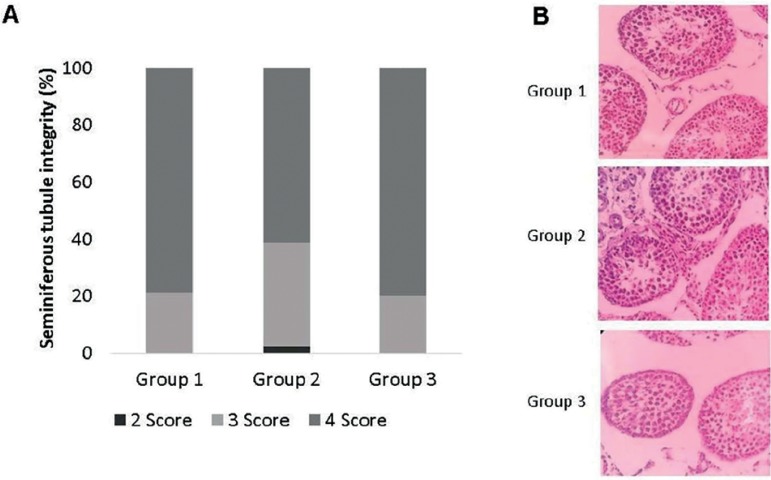
**A,** Ratio of seminiferous tubules per score in the three analyzed groups. **B,** Comparison between histological samples of fresh (Group 3) and vitrified (Groups 1 and 2) seminiferous tubules

Figure 2 depicts seminiferous tubules percentage per score. Histological analysis showed similar tubular integrity between fresh fragments from Group 3 and vitrified fragments from Group 1. Group 2 samples presented lower tubular integrity when compared to the other groups. The most found damage was detachment from the basement membrane in all groups (Table 1).

**Table 1:** Histological damage rate found by Group.

Group	Basement membrane detachment	Impossible Sertoli cells/spermatogonia differentiation	Hyalinization
1	70.58%	-	29.41
2	64.7%	8.8%	26.4
3	100%	-	-

### Autologous Heterotopic Grafting

**Figure 3:**
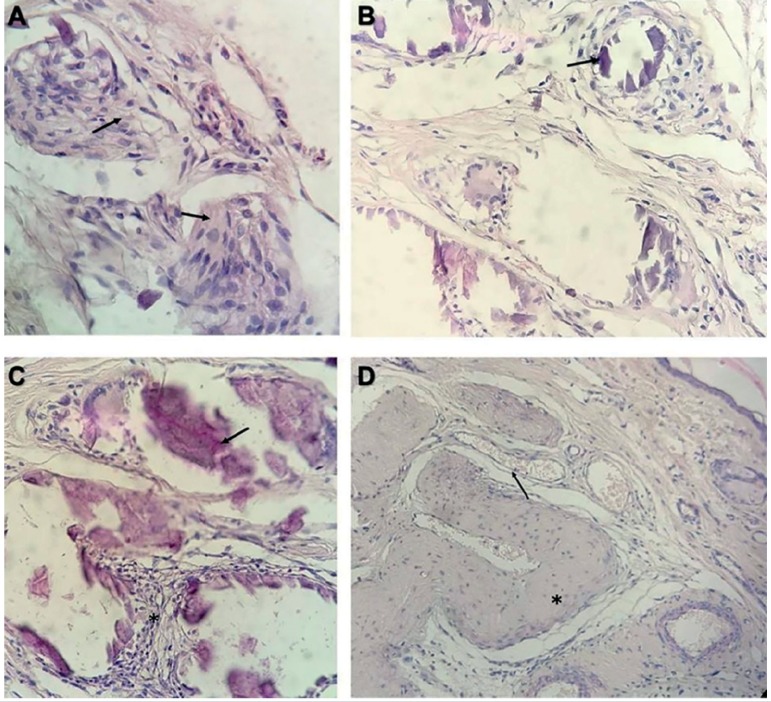
Sample graft representation 2 months after implant. **A,** presence of macrophages (arrows) near degenerated seminiferous tubules. **B,** presence of necrosis in degenerated tubules (arrow). **C,** coagulative necrosis (arrow) and lymphocytic interstitial infiltrate (asterisk). **D,** Presence of vascularization (arrows) surrounding seminiferous tubule (asterisk)

**Figure 4:**
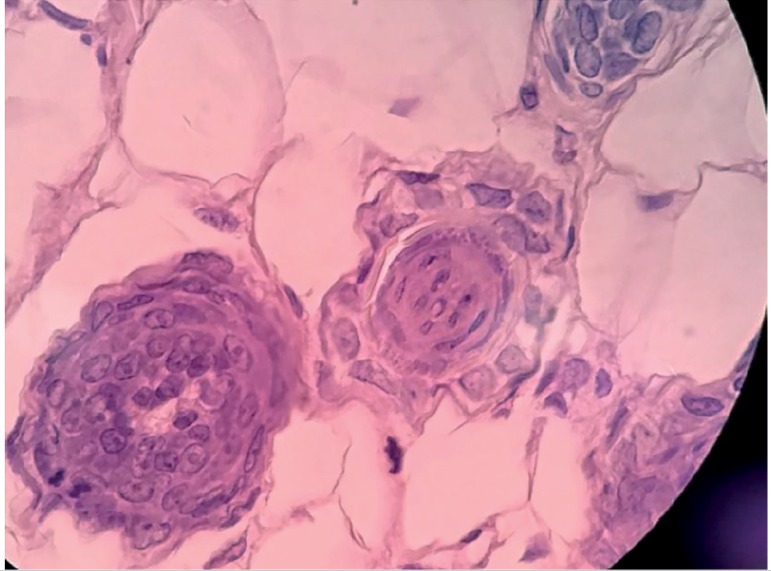
Sample graft representation showing seminiferous tubules in a Group 2 sample

Histological analysis from grafts of three groups (n=8) was performed, one ear per animal being evaluated. In all the groups, it was possible to observe the implant site through surgical scars; however, no fragment of testicular tissue or signs of inflammation were histologically observed in most samples. From the analyzed grafts, it was possible to identify in one sample from Group 2 the presence of degenerated seminiferous tubules with coagulation necrosis and signs of inflammatory processes, such as macrophages. In addition, signs of revascularization (blood capillaries) were found surrounding some of the remaining seminiferous tubules (Figure 3). In another sample from Group 2, intact seminiferous tubules could be found in the implant site (Figure 4).

## DISCUSSION

Two vitrification protocols were used in the present study to evaluate their efficiency in testicular cryopreservation. Vitrification is a fast method that has been shown to be effective in cryopreserving ovarian tissue and appears as an alternative to be explored since it preserves cells in the absence of ice crystals, thus removing the deleterious effects (Pegg, 2005).

Cellular viability recovery rates, verified by the Trypan blue dye exclusion test, demonstrated the efficacy of the testicular tissue vitrification protocols tested, and presented 72% and 59% of recovery for protocols 1 and 2, respectively. Protocol 2 presented inferior results in maintaining cell viability and this difference in viability recovery between the two tested protocols may be explained by the cryoprotectants concentration and the warming protocol, which were performed at different times. Lima *et al*. (2017) tested diverse cryoprotectant associations in cat prepubertal testicular tissue. Vitrification and DMSO/EG (20% and 24%) combination obtained the lowest quantification of nucleolar organizer regions of spermatogonia compared to other associations. The concentration of DMSO/EG association used by Lima *et al*. (2017) was similar to that of protocol 2 (20% DMSO, 20% EG), where lower viability results were also found.

The achievement of lower viability results using high cryoprotectant concentrations, such as DMSO may have been caused by toxic effects that the substance can induce, since it has been reported that DMSO may be capable of causing protein denaturation and cell membrane phospholipid bilayers destabilization (Arakawa *et al*., 2007). However, EG is not related to toxic effects and can be well tolerated even in high concentrations (Unni *et al*., 2012).

In a study about cryoprotectant toxicity, Unni *et al*. (2012) found that immature testicular tissue is twice as susceptible to damage, since adult tissue and cryoprotectants have shown a capacity to protect different cells from male germline. For instance, EG was shown to be the best of tested cryoprotectants to protect spermatocytes from damage, while DMSO showed greater ability to protect spermatogonia. DMSO has been reported in previous studies as the best isolated cryoprotectant for cryopreservation of immature murine testicular tissue in comparison with EG and Propanediol (Goossens *et al*., 2008; Milazzo *et al*., 2008), but viability rates were comparable with a DMSO/EG association.

Cryoprotectants efficacy in preserving certain cell types makes the search for associations of these substances a strategy to try to reduce the cryoprotectant's deleterious effects. The present study used a combination of DMSO and EG in both vitrification protocols with varying concentrations, finding that lower concentrations of cryoprotectants (15% of DMSO, 15% of EG) were more efficient in preserving cell viability. This result obtained on cell viability corroborates with the best protocol tested by Gurina *et al*. (2011) - slow cryopreservation of testicular tissue from adult Wistar rats using DMSO as cryoprotectant at a rate of 67.7% viability. Likewise, the results obtained were similar to those from Wu *et al*. (2012), who obtained 74% viability with slow cryopreservation of immature murine testicular tissue, using DMSO as the only cryoprotectant.

Gouk *et al*. (2011) also reported similar results, with murine immature testicular tissue vitrification, obtaining a cellular viability of 83%, found through flow cytometry and the use of cell markers to determine viable cells. The study conducted by the cited author showed remarkable differences in their protocol, since EG was used alone as a cryoprotectant and the samples were exposed to four increasing concentrations during vitrification. The samples were vitrified with the albuginea tunica being minimally penetrated by needles, whereas in this study the tunica was completely removed from the samples.

The carrier system may influence the viability rates and the support used in our vitrification protocols (1µL inoculation loop) diverged from several previous studies that used the straw-in-straw method (Gouk *et al*., 2011), aluminum foil floater (Baert *et al*., 2012) and open cryostraws (Curaba *et al*., 2011). It should be noted that the use of different cryoprotectants, maturity and tissue species, may interfere with cell viability rates after cryopreservation, since cryoprotectants have the ability to protect different cells of the male germ line, thus indicating that tissue maturity may be a determining factor for the choice of cryoprotectant used (Unni *et al*., 2012).

The cell viability found in this study was lower than the numbers found by Yamini *et al*. (2016), who presented a cell survival rate of 92% in an immature murine testicular parenchyma submitted to the vitrification protocol. Despite similarities in vitrification techniques that used the same cryoprotectants and the same method for cell viability assessment, some differences between the studies, such as fragment sizes, carrier system, and animal's species can be pointed out as the probable cause of different results concerning cell viability recovery.

It is agreed that the success of vitrification depends on factors such as sample size, carrier systems, concentration and time of exposure to the cryoprotectant (Liebermann *et al*., 2002). Among the variables, the size of testicular parenchyma fragments may have been one of the factors that contributed to the better results achieved by Yamini *et al*. (2016), since fragments of 0.5-1mm were used in comparison with those of this study, which were larger (3mm). It is known that the smaller the fragment size, the greater the chance of this being surrounded by liquid and not the nitrogen vapor, thus increasing cooling rates (Liebermann *et al*., 2002), favoring cryopreservation success.

Animal species should also be considered, since as demonstrated by Unni *et al*. (2012), testicular viability rates after cryopreservation may vary in samples from different species, even when using the same cryoprotectant solutions and protocols. The support method to vitrify the fragment may also interfere with the results, as they seek to increase cooling rates (Liebermann *et al*., 2002). Inoculation loops were used as support for testicular fragments in this study, while Yamini *et al*. (2016) used metal grids, which possibly provided a higher cooling rate to the tissue.

The histological analysis of the testicular fragments showed that Group 1 and Group 3 (fresh) tubules presented similar and equivalent tubular integrity, indicating little structural damage to the tissue when cryoprotectants in a lower concentration were used. This data corroborates those from Curaba *et al*. (2011), who observed structural similarities between fresh and vitrified tissue, also noticing that the greatest damage was the separation of the basement membrane. Group 2 presented tubules with higher structural damage when compared to other groups, in agreement with Lima *et al*. (2017), who found greater similar structural damage in samples exposed to comparable concentrations of DMSO/EG. Travers *et al*. (2011) used slow cryopreservation with propanediol for rat immature testicular tissue and found higher damage caused by epithelium gaps formation in its histological evaluation, besides those regarding the membrane detachment.

Immature murine testicular tissue vitrification protocols used by Hajiaghalou *et al*. (2016) differed from those applied in this study, because the samples were gradually exposed to combined (DMSO/EG) or isolated (EG) cryoprotectant concentrations. In their histological evaluation, they was also found basement membrane detachment as main damage, similarly to those of Groups 1 and 2. Protocol 1 obtained a good viability recovery rate, comparable to studies that used DMSO and EG as isolated cryoprotectants in both vitrification and slow cryopreservation protocols, preserving seminiferous tubules structure and demonstrating the possibility of using vitrification in testicular tissue cryopreservation.

Heterotopic transplantation showed signs of testicular structure disintegration and extensive seminiferous tubules degeneration, except for one sample where it was possible to identify intact seminiferous tubules. Obtained data corroborates with Makala *et al*. (2015) results, showing that when autologously transplanting testicular fragments (5mm) on the back of adult mice, there was an extensive seminiferous tubule degeneration 4 weeks after grafting and no intact seminiferous tubule present after 8 weeks.

When transplanting murine testicular tissue into the dorsum of nude castrated mice, Lim *et al*. (2014) showed an increase of sclerotic seminiferous tubules in the graft over time. Current data and previous studies (Lim *et al*., 2014; Makala *et al*., 2015) showed testicular tissue degeneration after heterotopic grafting. The absence of scrotal environment may have been one of the contributing factors for the testicular tissue degeneration, although the microenvironment and testicular niche were intact since fragments and non-cell suspensions were transplanted. Possible exposure to hyperthermic conditions at the implant site may also have contributed to fragment degeneration (Makala *et al*., 2015).

Luetjens *et al.* (2008) reported graft loss, after performing autologous transplant of testicular fragments of marmosets to ectopic sites using slow cryopreservation as the freezing technique; and they found the disappearance of transplanted tissue after 10 months. Even with visible implant site, no tissue or inflammation sign was found, as it happened to most of the current study grafts. In disagreement with Luetjens *et al.* (2008), fragment loss did not occur only to cryopreserved fragments, but also in freshly grafted fragments.

Fragment disappearance in our study may not be related to vitrification protocols, since no differences were seen between the groups with cryopreserved or fresh tissue. Factors that could contribute to graft disappearance are inflammatory response causing rejection and cell death, caused by ischemia (Jahnukainen *et al*., 2012).

Other attempts of testicular tissue ectopic grafting (Arregui *et al*., 2008; Makala *et al*., 2015) have demonstrated Sertoli cells survival despite seminiferous tubules extensive degeneration and sclerosis. Makala *et al*., (2015) also demonstrated Leydig cells degeneration after heterotopic transplantation, due to cellular hypoxia during the ischemic period, which is always present in the first moments after grafting. In our study, it was not possible to identify Leydig and Sertoli cells, hypothesizing that tubule hypoxia and sclerosis signs due to ischemia - that was evidenced in one of the grafts - may have occurred in all samples, making it impossible for somatic cells to remain in the implant sites. Dias *et al*. (2011) showed a rapid macrophage recruitment derived from peritubular monocyte differentiation when transplanting spermatogonia; and Jahnukainen *et al*. (2012) reported the presence of macrophages when transplanting testicular tissue from Rhesus monkeys to the back of nude mice. Interstitial infiltration of macrophages at the graft site was also demonstrated in our study, and macrophages may have been derived from peritubular monocytes or from blood recruitment, as it was possible to notice the presence of blood vessels surrounding grafted seminiferous tubules.

Testicular grafts that resulted in complete spermatogenesis were reported in orthotopic fresh testicular parenchyma transplants (Luetjens *et al*., 2008) and in cryopreserved and vitrified murine tissue (Baert *et al*., 2012). Kaneko *et al*. (2013) heterotopically grafted vitrified testicular tissue from pigs into nude mice and obtained spermatids, used to perform Intracytoplasmic Sperm Injection (ICSI), resulting in birth. However, literature data have shown negative results, as well as the present study, when autologous and heterotopic testicular tissue fragments were transplanted (Luetjens *et al*., 2008, Makala *et al.*, 2015).

Disparities in results when grafting testicular parenchyma shows that the implantation site is a determining factor since heterotopic transplantation does not enable efficient tissue revascularization. Orthotopic transplantation, in turn, provides high vascularization potential (Van Saen *et al*., 2009; Jahnukainen *et al.*, 2012), and should be considered as an alternative because it presents an ideal temperature, lower than that of the body, to support meiotic maturation and spermatogenesis (Luetjens *et al*., 2008).

Other implant sites, such as murine back musculature and kidney capsule, were able to reduce post-transplant graft hypoxia; but not in ovarian tissue (Soleimani *et al*., 2010), perhaps because of its high vascularization. However, testicular tissue has particularities that should be considered when choosing the graft site, in order to preserve the necessary environment conditions that enable spermatogenesis to occur.

## CONCLUSION

The present study demonstrated the efficacy of a vitrification protocol (DMSO 15%, EG 15%) in cryopreserving pre-pubertal testicular tissue of Wistar rats through a faster and more convenient method than slow cryopreservation. Cell viability and histological analysis of fragments showed little damage to vitrified tissue when cryoprotectants were used in a lower concentration.

Heterotopic transplantation could not preserve the structural organization of the testicular tissue, due to possible appearance of ischemia, degeneration processes and immunological responses, which should promote further research on a protective measure for the grafted tissue, such as biocompatible devices.
